# Quantitative study of the somatosensory sensitization underlying cross-modal plasticity

**DOI:** 10.1371/journal.pone.0208089

**Published:** 2018-12-05

**Authors:** Kenta Abe, Hiromu Yawo

**Affiliations:** 1 Department of Development Biology and Neuroscience, Tohoku University Graduate School of Life Science, Sendai, Japan; 2 Center for Neuroscience, Tohoku University Graduate School of Medicine, Sendai, Japan; University Paris 6, FRANCE

## Abstract

Loss of one sensory modality can cause other types to become more perceptive (cross-modal plasticity). To test the hypothesis that the loss of vision changes the perceptual threshold in the somatosensory system, we applied optogenetics to directly manipulate the afferent inputs involved in the whisker-barrel system using a transgenic rat (W-TChR2V4) that expresses channelrhodopsin-2 (ChR2) selectively in the large mechanoreceptive neurons in the trigeminal ganglion (TG) and their peripheral nerve terminals. The licking behavior of W-TChR2V4 rat was conditioned to a blue LED light cue on the whisker area while the magnitude and duration of light pulses were varied. The perceptual threshold was thus quantitatively determined for each rat according to the relationship between the magnitude/duration of light and the reaction time between the LED light cue and the first licking event after it. We found that the perceptual threshold was more significantly reduced than the control non-deprived rats when the rats were visually deprived at postnatal 26–30 days (P26-30, early VD group), but not at P58-66 (late VD group). However, the sensory threshold of a late VD animal was similar to that of a control. Our results suggest the presence of cross-modal plasticity by which the loss of vision at the juvenile period increased the sensitivity of the somatosensory system involved in the touch of whiskers.

## Introduction

Loss of one type of sensory modality can cause perceptual improvement of other sensory modalities. This process is called cross-modal plasticity [1–2]. For example, blind individuals compensate their lack of visual inputs by responding to somatosensory or auditory inputs with improved sensitivity and accuracy of perception. The brain can adapt to sensory deprivation in one modality by increasing plasticity and retuning neuronal circuits in other remaining modalities. Therefore, cross-modal plasticity of different sensory systems may require a coordinated shift of attention from the deprived to the intact sense [3].

The perceptual threshold is the weakest stimulus that an animal can detect and create perception, and should be under the regulation of neural computation in the central nervous system (CNS). Therefore, the loss of one modality may affect the threshold in the others. For example, the rodent vibrissae sensory system has been one of the well-documented research models to investigate the effects of visual deprivation (VD) on the somatosensory perception [4–7]. Indeed, when the rat was visually deprived at birth, it performed the maze task, which is dependent on the tactile sense using whiskers, better than the non-deprived ones [8]. The reaction time is a useful parameter to evaluate the decision making, which is dependent on the strength and duration of the stimulus. When the stimulus strength is large, the reaction time becomes small, and *vice versa* [9]. These data indicate that the reaction time should be useful to estimate the perceptual threshold. However, there remain technical difficulties in animal models of controlling the pattern and magnitude of whisker inputs quantitatively to measure the reaction time precisely.

To overcome this, we used optogenetics to make afferent inputs involved in the whisker-barrel system directly by light. Previously, we generated a transgenic line of rat, W-TChR2V4, that expresses channelrhodopsin-2 (ChR2) in the large mechanoreceptive neurons but not in the nociceptive ones in the trigeminal ganglion (TG) [10–13]. As the whisker follicles are densely innervated by ChR2-positive mechanoreceptive nerve endings, the irradiation with blue light evoked electrical and fMRI responses in the barrel field of the contralateral somatosensory cortex [14]. Using this rat model, we recently demonstrated that optogenetic stimulation of the whisker area is a robust cue for decision making under head-fixed conditioning tasks [15]. Therefore, the behavioral outputs should be quantitatively measured while controlling the sensory inputs precisely by light. We found that the perceptual threshold was significantly reduced when the rats were visually deprived at postnatal 4 weeks (P26-30), but not at later (P58-66). It is suggested that the sensitivity to somatosensory inputs would be changed by the loss of visual inputs in a manner dependent on the developmental stages of the animal.

## Materials and methods

### Animals

All animal experiments were carried out in accordance with the animal experiment protocol approved by Tohoku University Committee for Animal Experiments and in accordance with the guidelines for Animal Experiments and Related Activities of Tohoku University as well as the guiding principles of the Physiological Society of Japan and the National institutes of health (NIH), USA. The number of animals in this study was kept to be minimal and, when possible, all animals were anesthetized to minimize their suffering. Animals had access to food and water *ad libitum* and were kept under a 12 hour light-dark cycle. Transgenic rats expressing ChR2-Venus under the control of thy1.2 promoter in the central and peripheral nervous systems (W-TChR2V4) [NBRP#0685, http://www.anim.med.kyoto-u.ac.jp/NBR/Default.aspx] were used throughout the experiments.

#### Visual deprivation

Two groups of W-TChR2V4 rats were visually deprived bilaterally at either postnatal day 26–30 (P26-30, early VD group) or P58-66 (late VD group). Briefly, under anesthesia with a mixture (1 ml/kgBW) of ketamine (50 mg/ml, Daiichi Sankyo Co. Ltd., Tokyo, Japan) and xylazine (xylaize hydrochloride, 10 mg/ml, Sigma-Aldrich, St. Louis, MO, USA), a small incision was made with surgical scissors in the conjunctiva beginning inferior to the globe and around the eye temporally. In order to protect the underlying extraocular muscles from injury, care was taken so as not to cut the tissue too deeply. When the posterior face of the globe was exposed, the optic nerve was amputated under visualization with minimal damage to the vasculature [16].

#### Head-plate implantation and adaptation

All experimental rats were implanted with a head-plate under ketamine-xylazine anesthesia (1 ml/kgBW). A head plate (CFR-1, Narishige, Japan) was surgically attached to the skull of each rat with tiny anchor screws (M1.4×3) and dental resin cement (Super Bond C&B, Sun Medical, Moriyama City, Japan), while its body temperature was maintained at 37°C using a homeothermic heating pad. Three-to-four days after recovery from the surgery, the rats were deprived of drinking water over 48 hours in their home cage, where food was available *ad libitum*, and were adapted to a stainless steel cylinder in which they were inserted during the following behavioral experiments [17].

### Optical system

All whiskers as well as the intervibrissal fur of the right side were trimmed off from the snout of the rat. The right whisker area was irradiated by the light pulse generated from a blue LED (470 nm, LXML-PB01-0040, Philips Lumileds Lighting Co. San Jose, CA, USA). The leakage of LED light was minimized by setting it close to the snout (~5 mm). The duration and intensity of the LED pulse were variably regulated by an LED driver (FCS-0470-000, Mightex Systems, Toronto, Canada) and a computer. The power of light was measured at 5 mm distance using an optical power meter (8230E, ADCMT, Saitama, Japan).

### Behavioral test

**Fig 1:**
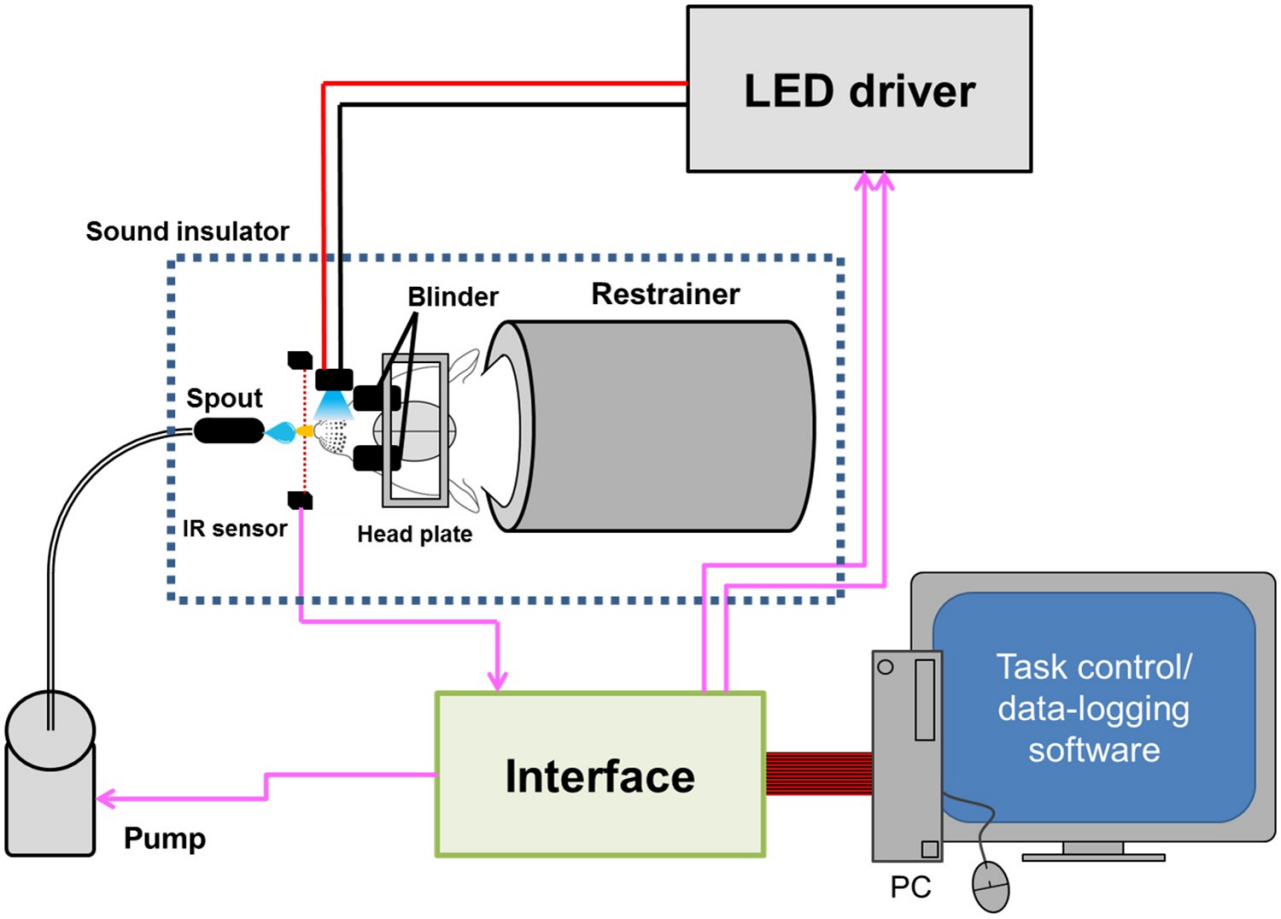
Behavioral test system. The head of the fully awake rat was fixed with a head plate in a stereotaxic frame while its body was in a restrainer. The lick was detected by the infrared (IR) sensor in front of its mouth. When the lick was judged to be successful, a drop of sweet water was pumped out from the spout as a reward. The stimulus to the whisker area was made by a pulse of blue LED light, the on-off function of which was controlled by the LED driver. These devices were under control of a software through an interface while logging the sensor signal. The rat was isolated from environmental sound and both eyes were masked to prevent the pump sound or leaked light from being utilized as a learning cue.

The behavioral test system (Fig 1) consisted of a stereotaxic frame (Model 900, David Kopf Instruments, Tujunga, CA, USA) where the awake rat was head-fixed with its head plate, a liquid feed pump, an LED driver (FCS-0470-000, Mightex Systems, Toronto, Canada), a computer, an interface controller device and a task control software (TaskForcer, O’HARA Co, Ltd., Tokyo, Japan). A spout was set in front of the rat’s mouth and water containing 0.1% saccharin (5 μl) was pumped out as a reward. When the rat licked the nozzle, it was detected by the infrared (IR) radiation sensor. During the experiment, the rat was equipped with eye masks and isolated from the environmental sound to prevent the leaked light or the pump sound from being utilized as a learning cue. Each training session was 0.5-*h* series of tasks sequentially applied with an inter-task interval (ITI) of either 20 ± 5 s. The ±5-s variability of ITI was given randomly so that the rat could not expect the timing of rewards.

### Data analysis

All data were logged by the manufacturer’s software (Operant Task Studio, O’HARA Co, Ltd.), analyzed off-line by home-made programs and expressed mean ± SEM (number of animals). The statistical evaluation was conducted using one-way ANOVA Turkey-Kramer post hoc test (*p < 0.05, **p < 0.01). It was judged as statistically insignificant when p > 0.05.

## Results

### Establishment of the optogenetic conditioning

**Fig 2:**
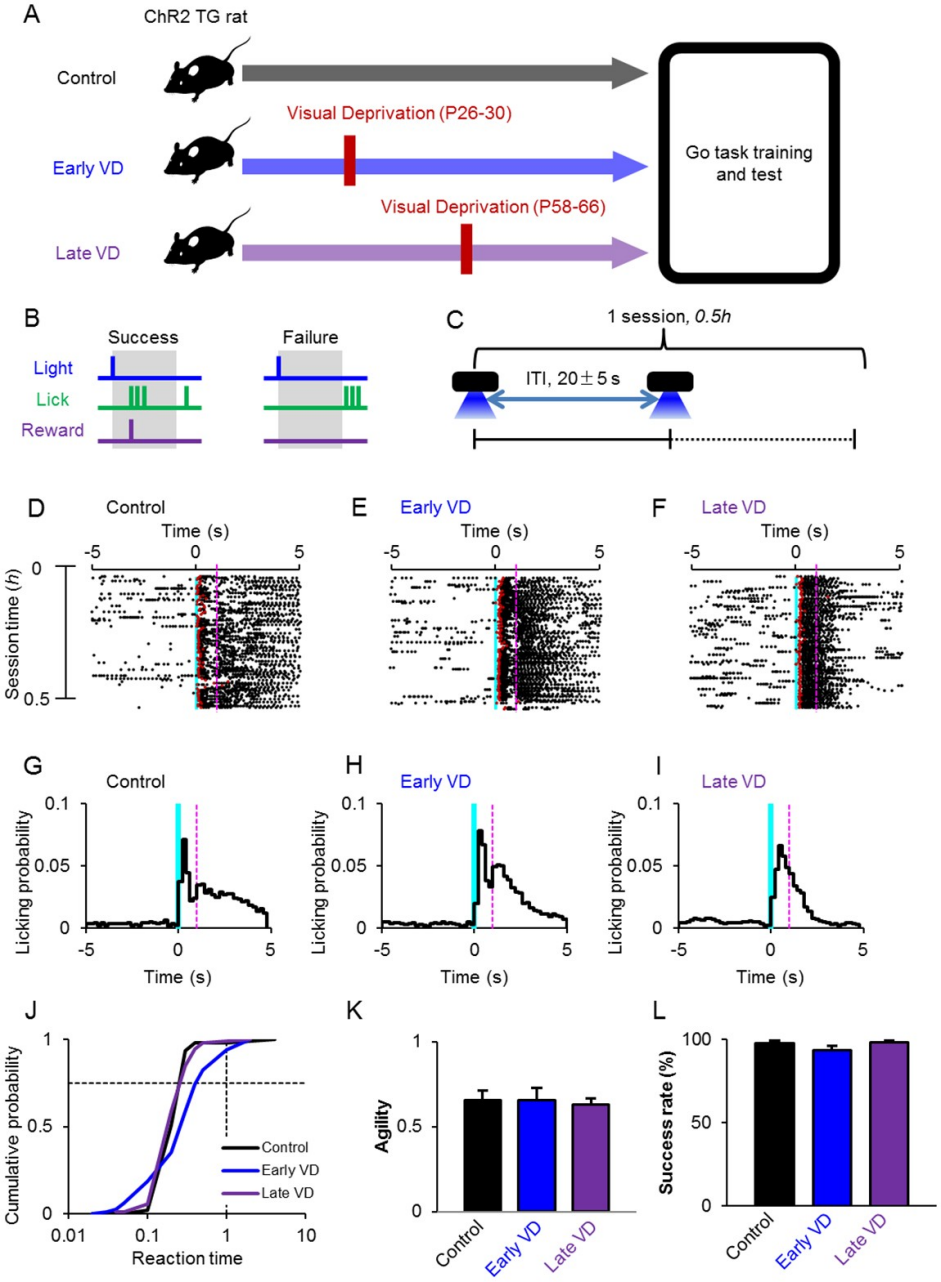
Establishment of the optogenetic conditioning. **A**, Experimental protocol. **B**, Go task paradigm. The rat was rewarded when it licked the nozzle within the 2-s time window (success, left), but not when it did not lick within the time window (failure, right). **C**, The LED stimulus was repeated with 20 ± 5 s inter-trial interval (ITI), one conditioning session was 0.5 *h*. **D-F**, Sample raster plots of the licking events 5-s before and after the onset of the LED stimulus cues (blue vertical line), the control (D), early VD (E), late VD (F) rats, respectively, while the red marks indicate the rewarded lick. **G-I**, Histograms of the licking probability before and after 5 s of the onset of LED stimulus cues (blue vertical line) of the same rats shown previously (D-F). **J**, Cumulative probability plots of the reaction time. The vertical broken lines was drawn at 1 s while the horizontal ones at 0.75. **K**, Summary of the agility: the control (n = 9), early VD (n = 10) and late VD (n = 8) groups. One-way ANOVA Turkey-Kramer post hoc test: *p* = 0.99 (the control vs. early VD); *p* = 0.95 (the control vs. late VD) and *p* = 0.95 (early vs. late VD). **L**, Summary of the success rate: the control (n = 9), early VD (n = 10) and late VD (n = 8) groups. One-way ANOVA Turkey-Kramer post hoc test: *p* = 0.3 (the control vs. early VD); *p* = 0.99 (the control vs. late VD) and *p* = 0.27 (early vs. late VD). In A-F, each magenta broken line was drawn at 1 s after the cue stimulus.

Three groups of the W-TChR2V4 rats were trained for a Go-task paradigm conditioned to the photostimulation on the whisker area: the early VD group in which the animals were visually deprived at P26-30, the late VD group in which the animals were visually deprived at P58-66, and the control group without VD (Fig 2A). A Go-task training paradigm was so designed to reward the saccharin water once when the rat licked the nozzle within 2 s after the onset of a brief LED stimulus cue (Fig 2B, success). However, the rat was not rewarded when it did not lick the nozzle within this judgment time window (failure). After training that consisted of the Go task for 0.5 *h* with an inter-trial-interval (ITI) of 20 ± 5 s (Fig 2C), a Go-task conditioning test was made with salient photostimulation (12.4 mW/mm^2^ for 50 ms). In all groups, every animal was successfully conditioned to lick the nozzle synchronously with the blue light cue after several training sessions (Fig 2D–2F) [15]. Indeed, the licking probability was increased after the LED stimulus cue with a short delay (Fig 2G–2I). When the reaction time was measured between the onset of the light cue and the first licking event, it distributed mostly less than 1 s in all groups (Fig 2J). Previously, the distribution of the reaction time was quantitatively represented by the time when the cumulative probability reached to 0.75 (the third quartile of reaction time distribution, *RT*_75_) and the logarithmic reciprocal of this value, -log (*RT*_75_), which we referred to “agility”, was shown to be a quantitative indication of the reaction speed of an animal [15]. The agility is positive when 75% of the cue trials were accompanied by the first licking events within 1 s and is negative thereafter. Indeed, we observed previously that the agility was almost negative throughout sessions of any rats in the wild-type and unpaired groups whereas it changed from negative to positive with the progress of the training sessions for the Go-task conditioning of W-TChR2V4 rats [15]. As shown in Fig 2K, the agility was indeed positive after establishment of the Go-task conditioning for every animal without significant differences among three groups. The success rate, the chance of licking within the judgment time window was also measured for the last testing session (Fig 2L). It was over 50% for every animal without significant differences among three groups.

We also performed three kinds of sham test: (1) wild type rats paired with reward (WT/reward), (2) conditioned W-TChR2V4 rats without LED light cue (ChR2/no light) and (3) naïve W-TChR2V4 rats without reward (ChR2/no reward). In each case, the licking events occurred less synchronized to the LED stimulus cue, the agility was negative and the success rate was less than chance level (50%) (S1 Fig). Therefore, the influences such as “faint perception” by the visual stimulus cue and the spontaneous licking behavior for the sampling reward were negligible.

### Near-threshold stimulus task

**Fig 3:**
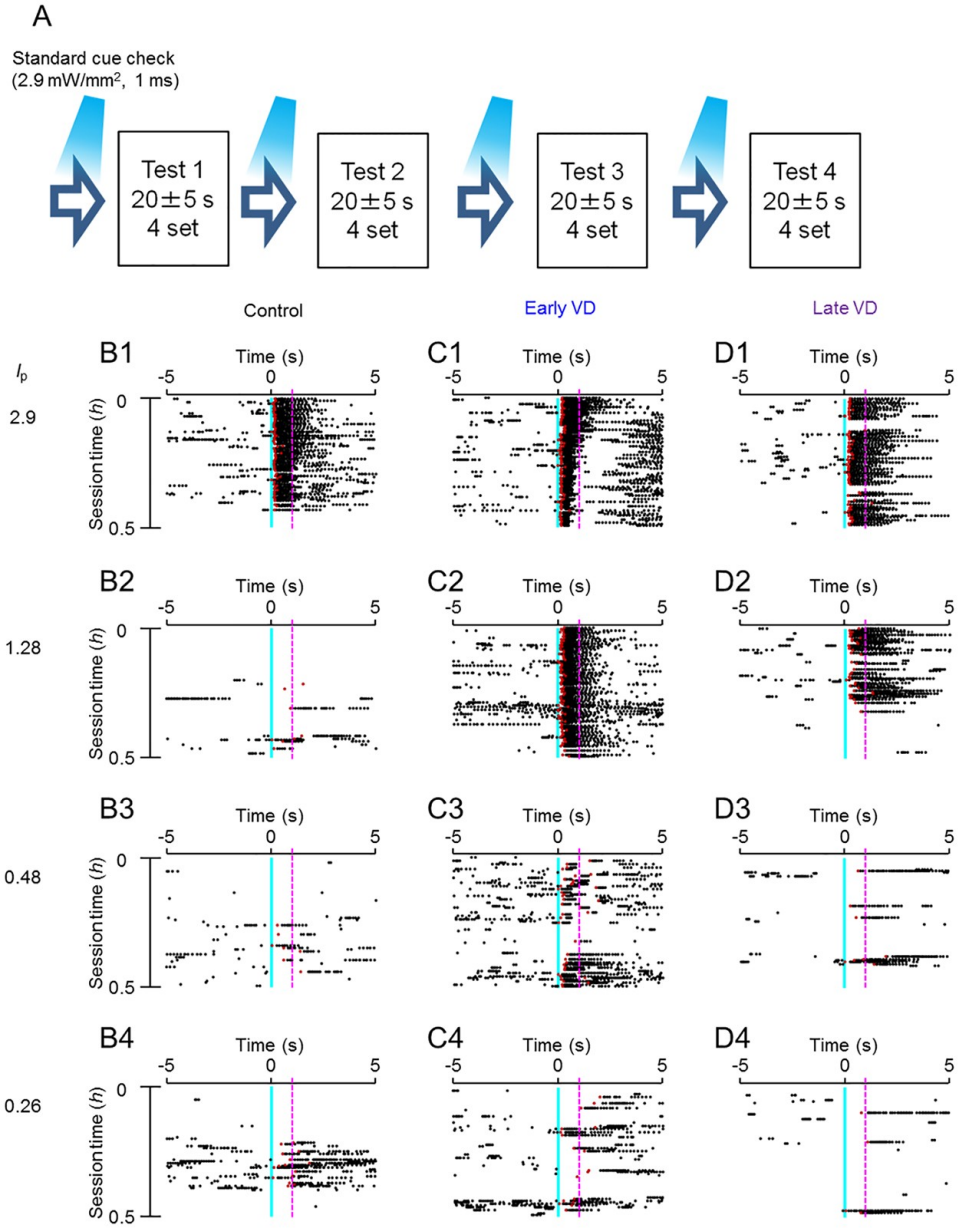
Near-threshold task performance under test 1 (fixed LED duration at 1 ms). **A**. Schema of the near-threshold task. **B1-4**, Sample raster plots of the licking events of a control rat before and after 5 s of the onset of LED cues (blue vertical line) with a fixed LED pulse duration (*t*_p_, ms) at 1.0 while decreasing the power (*I*_p_, mW/mm^2^) from 2.9 (B1), 1.28 (B2), 0.48 (B3) to 0.26 (B4). **C1-4**, Similar to B1-4, but of an early VD rat. **D1-4**, Similar to B1-4, but of a late VD rat. In B1-4, C1-4 and D1-4, each magenta broken line was drawn at 1 s after the cue stimulus.

After establishment of the conditioning to the LED cue on the whisker area, 4 kinds of test were made for rats to determine the perceptual threshold for the somatosensory input (Fig 3A); Test 1, a fixed LED pulse duration (*t*_p_, ms) at 1.0 with decreasing power (*I*_p_, mW/mm^2^) from 2.9 to 1.28, 0.48 and 0.26 (Fig 3); Test 2, a fixed *t*_p_ at 0.6 with decreasing *I*_p_ from 2.9 to 1.28, 0.48 and 0.26 (S2 Fig); Test 3, a fixed *I*_p_ at 2.9 with deceasing *t*_p_ from 1.0 to 0.5, 0.2 and 0.1 (S3 Fig) and Test 4, a fixed *I*_p_ at 1.56 with deceasing *t*_p_ from 1.0 to 0.5, 0.2 and 0.1 (S4 Fig). Before each test the standard blue LED stimuli (2.9 mW/mm^2^, 1 ms) were given repetitively for a few minutes to confirm if they were followed by the synchronized licking events with short reaction time. Although the licking events were synchronized to the LED cue of the standard *I*_p_, they became less synchronized with the reduction of *I*_p_ for every rat in either control (Fig 3B, panels 1–4), early VD (Fig 3C, panels 1–4) or late VD group (Fig 3D, panels 1–4) during test 1.

**Fig 4:**
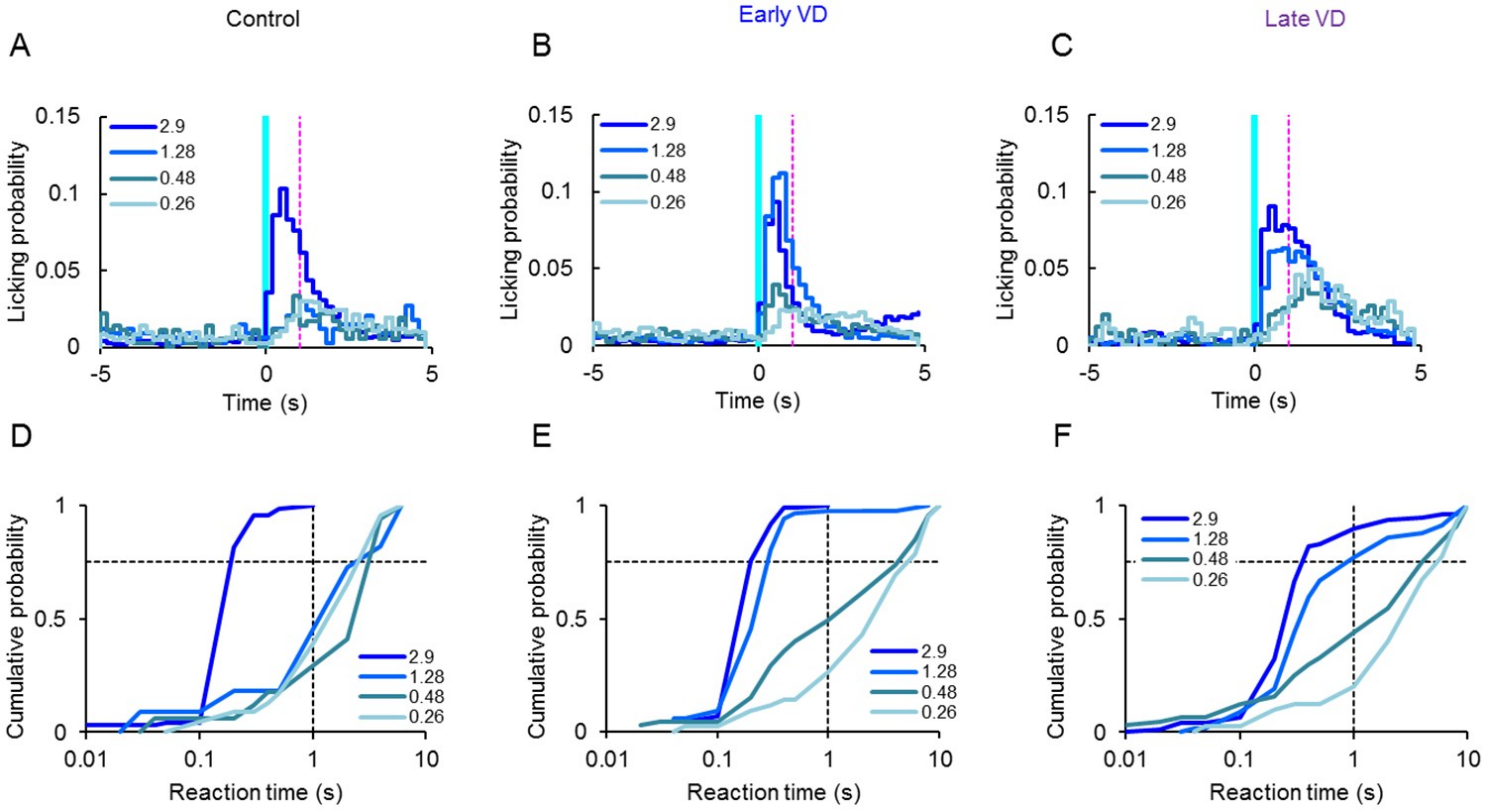
Licking probability and time to first lick under test 1. **A-C**, Histograms of licking probability before and 5 s after the onset of the LED stimulus cue (blue vertical line) of the same rat, the control (A), the early VD (B), the late VD (C). Each magenta broken line was drawn at 1 s after the cue stimulus. **D-F**, Cumulative probability plots of the reaction time of the same rats shown previously. The vertical broken lines was drawn at 1 s while the horizontal one at 0.75.

The desynchronization was accompanied by a reduction of the licking probability immediately after the cue stimulus (Fig 4A–4C). Although the reaction time between the onset of the LED cue and the first licking event was mostly distributed to less than 1 s for the large *I*_p_ (eg. 2.9 mW/mm^2^), it distributed rather evenly across the test period when *I*_p_ was decreased for every rat in either the control (Fig 4D), the early VD (Fig 4E) or the late VD group (Fig 4F).

Similar responses were observed during test 2. The licking event was less synchronized to the cue of smaller *I*_p_ for every rat in the control (S2A Fig), early VD (S2B Fig) and late VD groups (S2C Fig). The desynchronization was also accompanied by a reduction of the licking probability immediately after the cue stimulus (S2D–S2F Fig). The reaction time distributed less in 0–1 s period with the reduction of *I*_p_ for every rat in either the control (S2G Fig), the early VD (S2H Fig) or the late VD groups (S2I Fig).

The licking event was less synchronized to the cue of smaller *t*_p_ (test 3) for every rat in the control (S3A Fig), early VD (S3B Fig) and late VD groups (S3C Fig), as shown in the distribution of the licking probability (S3D–S3F Fig). The reaction time distributed less in 0–1 s period with the reduction of *t*_p_ for every rat in either the control (S3G Fig), the early VD (S3H Fig) or the late VD groups (S3I Fig).

Similar results were observed during test 4. The licking event was less synchronized to the cue of smaller *t*_p_ for every rat in the control (S4A Fig), early VD (S4B Fig) and late VD groups (S4C Fig) as shown in the distribution of licking probability (S4D–S4F Fig). The reaction time distributed again less in 0–1 s period with the reduction of *t*_p_ for every rat in the control (S4G Fig), early VD (S4H Fig) and late VD groups (S4I Fig). Finally, we tested the effects of satiety on the agility and performed the salient stimulus task for a long period of time (0.5 *h*, 16 sets). We confirmed that there was no decrease of the agility due to satiety and it was maintained in the positive zone (S5 Fig).

### Estimation of perceptual threshold

**Fig 5:**
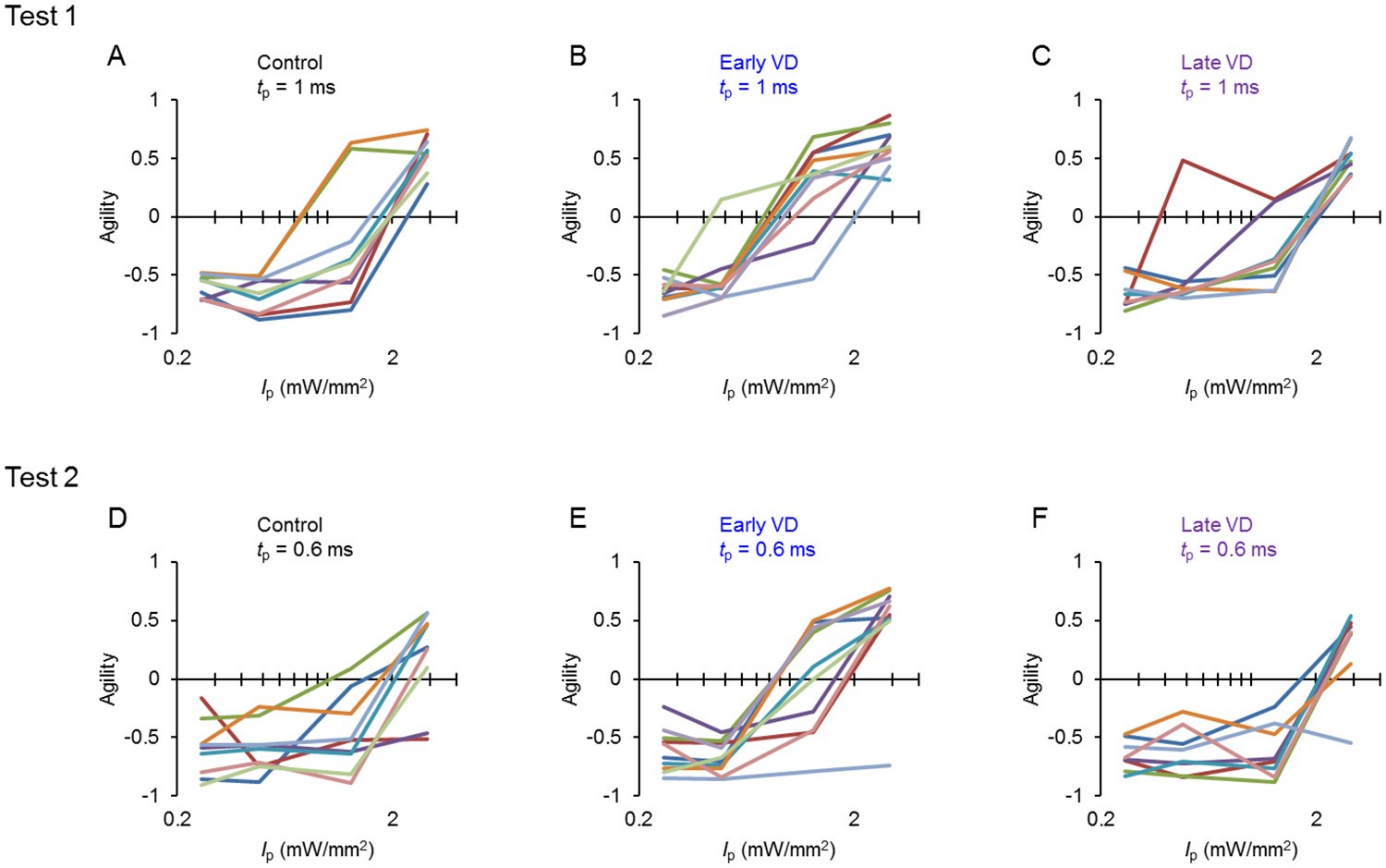
The shift of agility during test 1 and test 2. **A-C**, The changes of agility during test 1, each color indicates an individual rat in the control (A, n = 9), early VD (B, n = 10) and late VD (C, n = 8) groups. **D-F**, Similar to A-C, but during test 2, the control (D, n = 10), early VD (E, n = 9) and late VD (F, n = 8) groups.

During these tests the distributional change in the reaction time was represented as the change of agility. For example, during test 1 for every rat of the control (Fig 5A), early VD (Fig 5B) and late VD group (Fig 5C), the agility was positive at the standard *I*_p_ (2.9 mW/mm^2^) but became reduced to negative with the reduction of *I*_p_. Indeed, the agility was usually negative for every rat that had not been conditioned for the LED cue whereas it became positive with the establishment of conditioning [15]. We thus defined the perceptual threshold as the *I*_p_ evoking a behavioral response that changed the sign of agility from negative to positive. Practically, it was estimated from the agility (y)-*I*_p_ (x) relationship as the x-interception of a log-linear line connecting the point of negative agility with the largest *I*_p_ and that of positive agility with the smallest *I*_p_ (S6 Fig). When the agility was negative even at the standard *I*_p_, it was adopted as the perceptual threshold. Similarly, the perceptual threshold was estimated from the agility-*I*_p_ relationship during test 2 for every animal of the control (Fig 5D), the early VD (Fig 5E) and the late VD groups (Fig 5F).

**Fig 6:**
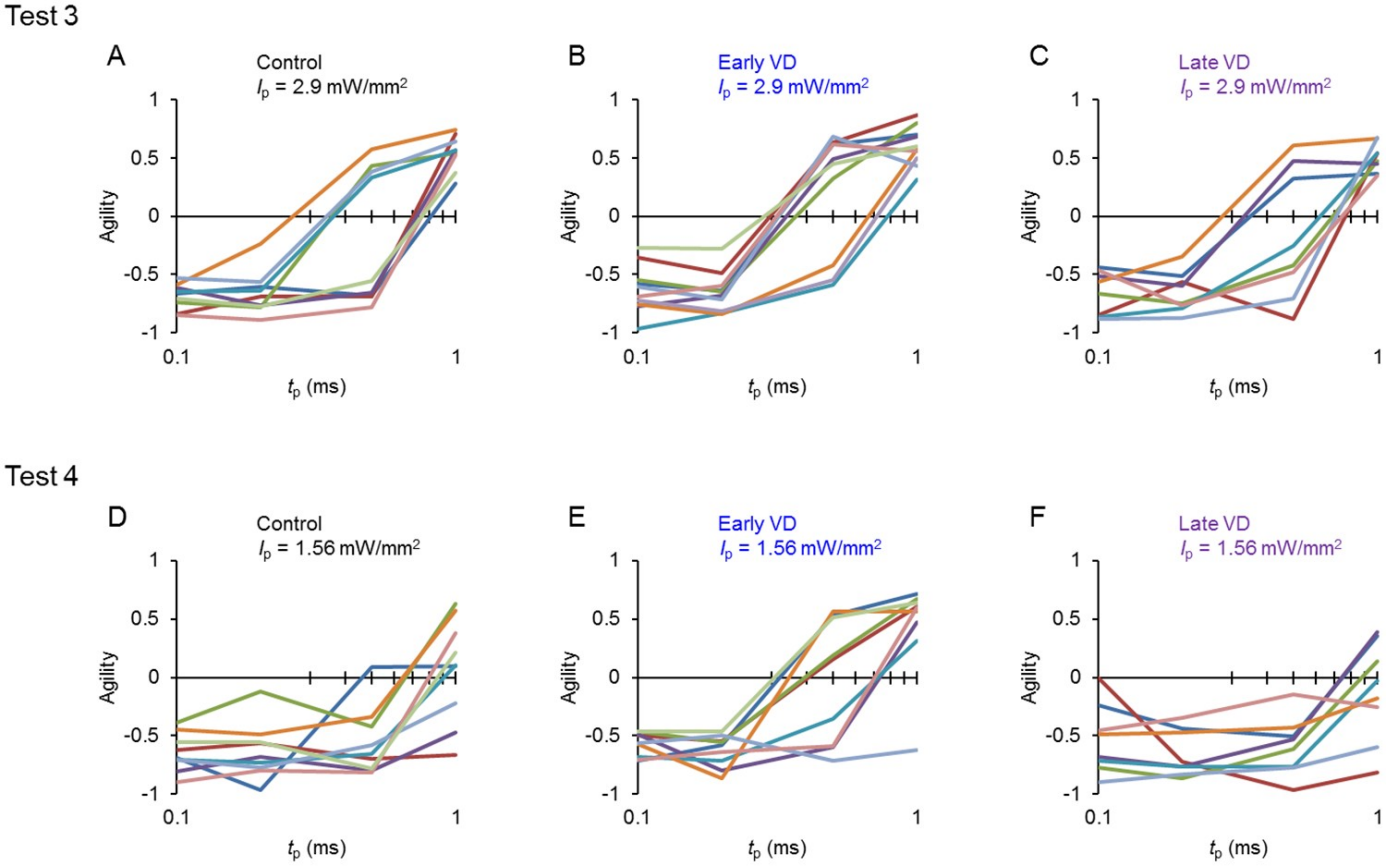
The shift of agility during test 3 and 4. **A-C**, The changes of agility during test 3, with each color indicating the individual rat in the control (A, n = 9), early VD (B, n = 10), late VD (C, n = 8) groups. **D-F**, Similar to A-C, but during test 4, the control (D, n = 9), early VD (E, n = 9), late VD (F, n = 8). One of 10 early VD rats was not included because its head plate was dislocated during test 4.

On the other hand, the perceptual threshold (ms) was estimated from the agility-*t*_p_ relationship during test 3 (Fig 6A–6C) and 4 (Fig 6D–6F) for every animal of the control, the early VD and the late VD groups. The age of the ChR2 rat used for the experiment is described (Table 1).

**Table 1:** W-TChR2V4 rats used for the data collection.

Control				Early VD				Late VD			
ID	sex	VD	Test	ID	sex	VD	Test	ID	sex	VD	Test
116	♂	-	P78	130	♂	P30	P76	143	♀	P62	P103
125	♀	-	P118	131	♂	P30	P76	144	♀	P62	P104
147	♂	-	P70	132*	♂	P30	P79	146	♀	P65	P107
148	♂	-	P71	133	♂	P27	P74	163	♂	P65	P94
158	♂	-	P81	135	♂	P26	P76	164	♂	P65	P94
159	♂	-	P81	136	♀	P30	P96	166	♀	P66	P121
160	♀	-	P109	137	♀	P30	P98	185	♀	P58	P104
183	♂	-	P92	139	♀	P27	P105	186	♀	P58	P104
189	♂	-	P70	140	♂	P26	P99				
				181	♂	P28	P92				

*(ID132), test 4 data was not collected because the head plate of this rat was dislocated during the experiment.

**Fig 7:**
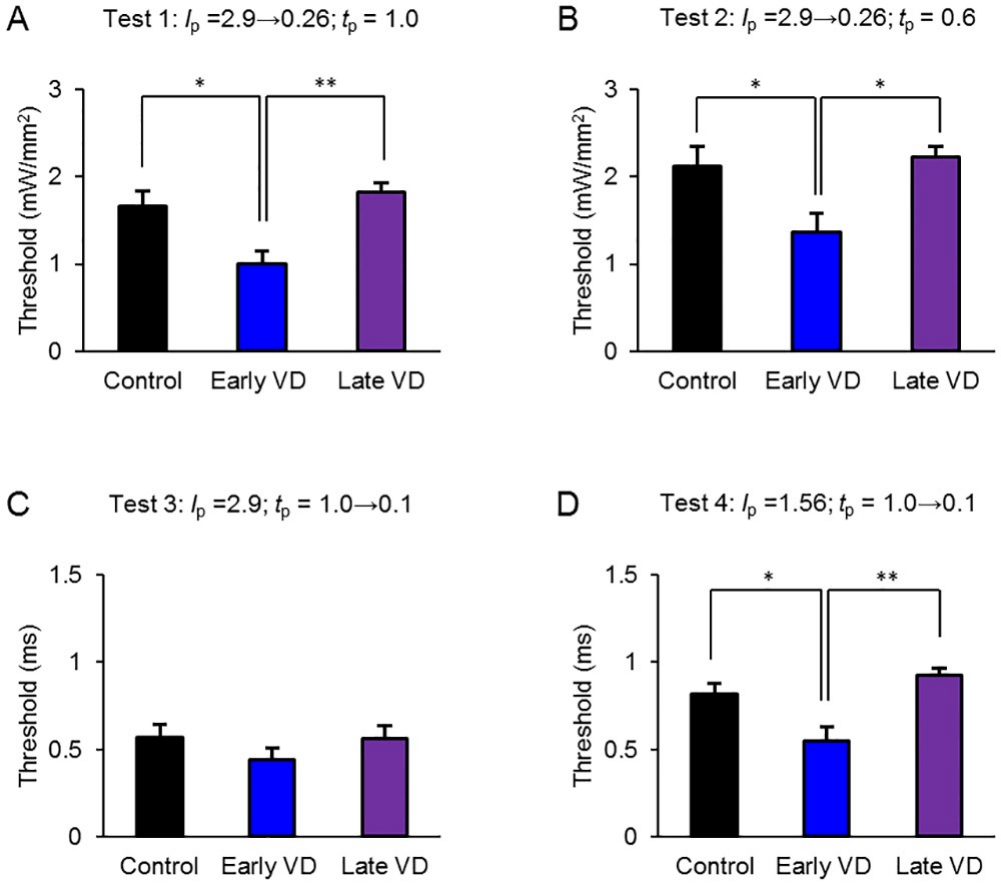
Comparison of the perceptual threshold. **A**, Summary of the thresholds by test 1, the control (n = 9), the early VD (n = 10) and the late VD (n = 8) groups, respectively from left to right: *p* = 0.012, the control vs. early VD; *p* = 0.75, the control vs. late VD and *p* = 0.0026, early VD vs. late VD. **B**, Similar to A, but by test 2: *p* = 0.027, the control vs. early VD; *p* = 0.93, the control vs. late VD and *p* = 0.014, early VD vs. late VD. **C**, Similar to A, but by test 3: *p* = 0.41, the control vs. early VD; *p* = 0.99, the control vs. late VD and *p* = 0.45, early VD vs. late VD. **D**, Similar to A, but by test 4: *p* = 0.021, the control vs. early VD; *p* = 0.51, the control vs. late VD and *p* = 0.0018, early VD vs. late VD. *, *p* < 0.05, **, *p* < 0.01, one-way ANOVA Turkey-Kramer post hoc test.

Based on the above results the distribution of the perceptual threshold appeared to be different between the early VD group and the others. Indeed, the perceptual threshold of the early VD group was significantly smaller than the control and late VD groups for test 1, 2 and 4 (Fig 7A, 7B and 7D). However, the difference was not significant for test 3 (Fig 7C). These date indicated that the perceptual threshold was more significantly reduced than the control non-deprived rats when the rats were visually deprived at P26-30 (early VD group), but not at P58-66 (late VD group).

## Discussion

In the present study, we evaluated the perceptual threshold of the somatosensory inputs quantitatively on the whisker-tactile detection task using optogenetics of the W-TChR2V4 rat, which expresses ChR2 in the mechanoreceptive neurons in the TG. In the present experiments, we used a light pulse with small *t*_p_ (0.1–1 ms) as a conditioning cue to measure the reaction time precisely. In the ChR2 expressing neuron, the light-evoked depolarization depends on some parameters such as the pulse duration, the light intensity and the membrane time constant. In this case, although *t*_p_ was small, its standard *I*_p_ was large (2.9 mW/mm^2^). In addition, we irradiated a wide area in the whisker-pad for the LED stimulus cue to activate many TG neurons at once. Further, the TG neuron’s membrane time constant is fast: a 1 ms mechanical stimulation can evoke neural activity and reward-related behavior [18]. Therefore, the light-evoked somatosensory perception can be formed even for a short time pulse. Less number of activated afferent inputs is also expected with the reduction of *I*_p_ or *t*_p_.

We revealed, for the first time, that the perceptual threshold was significantly reduced in the early VD group, indicating the presence of cross-modal plasticity by which the loss of vision increased the sensitivity of the somatosensory system involved in the touch of whiskers. Because the reaction time between the blue LED cue and the first lick was dependent on the intensity and duration of the stimulation pulse, it should be an indication of how the stimulus strength affects the decision making [19–20]. The distributional change of the reaction time for a 0.5 *h*-session of the Go task was evaluated using a new parameter, referred to as “agility”, which was usually negative for animals unconditioned to the LED cue but became positive when conditioned [15]. Indeed, the agility was a function of both the power of light (*I*_p_) and the duration (*t*_p_) of the LED pulse. This enabled us to estimate the perceptual threshold as an *I*_p_ or a *t*_p_ where the agility changed its sign from negative to positive. The perceptual threshold of early VD animals was significantly smaller than that of control or late VD animals during the near-threshold test 1 and 2, in which the *I*_p_ was changed with a fixed *t*_p_, as well as during test 4, in which the *t*_p_ was changed with a fixed *I*_p_. However, the perceptual threshold of late VD animals was similar to that of control animals. The cross-modal plasticity of the somatosensory system appeared to be dependent on the age of the loss of vision and the reduction of perceptual threshold could be manifest when it occurred early in the development. In test 3, no significant difference of the perceptual threshold was shown despite the same series of *t*_p_ as test 4. It is possible that the whisker-tactile perception is not a simple function of *I*_p_ and *t*_p_, but is dependent on the stimulus pattern. When the salient stimulus was given, the significant difference in the success rate and reaction time disappeared in each animal group, indicating that there was no change in the minimal reaction time (or the maximal agility).

The reaction time is more than the simple sum of the time spent for sensory input and motor output (sensory-guided behavior) and is regarded as a direct reflection of the time for decision [20] and is affected by cognitive functions such as attention [21–22]. However, there is no corresponding increase in the level of sensory inputs from the peripheral organs [23–24]. Cross-modal plasticity allows the same bottom up sensory inputs to more strongly influence the activity of the cortical neurons, and VD decreases the neural threshold for sound intensity in A1 L4 neurons [24] and shapes the neuronal receptive fields in A1 [25] and barrel [26].

At the molecular/cellular level, the VD increases the extracellular serotonin in the barrel cortex and facilitates the synaptic delivery of AMPA-type glutamate receptors (AMPARs) at L4-2/3 synapses in the barrel cortex of juvenile rats [26]. Additionally, transient strengthening of excitatory synapses at L4-2/3 in the barrel cortex could trigger an enhancement of the inhibitory inputs to the neighboring barrel, and sustained lateral inhibition can maintain the sharpening of whisker-barrel map [27]. The functional architecture of the adult neocortex is presumed to be organized by the excitatory-inhibitory balance [28]. Probably, VD is involved in this balance. Thus, this should also be observed when VD is initiated in adulthood [24]. However, the majority of these studies focused on the deprived cortex as the main substrate for functional and behavioral compensation that accompanies early sensory loss [1]. Being consistent with these studies, we did not find a significant difference between the late VD and control groups. Alternatively, the induction of VD-mediated cross-modal plasticity could be dependent on the developmental stages. On the other hand, it should be noted that the perceptual threshold varied depending on the intensity and duration in the LED stimulus cue. If we would change these parameters, we could find a significant difference between the late VD and the control. It is also possible that the perceptual threshold could be decreased by the increase of some topdown inputs into the primary somatosensory cortex (S1). For example, the optogenetic depolarization of apical dendrites facilitated the generation of dendritic Ca^2+^ spikes in the later 5 pyramidal cells in the barrel cortex [9]. The topdown inputs to the superficial cortical layers would be thus amplified [29] with a reduced perceptual threshold in the S1 [9]. These hypotheses are consistent with the notion that the barrel cortex of early VD rats would become more sensitive to the whisker inputs than the non-VD ones. However, it should be investigated whether the same mechanism could explain the difference between early and late VD animals. Other molecular and circuitry mechanisms could also be involved in the process of perceptual decision making and determine the reaction time.

## Conclusions

A numerical analysis using optogenetics enabled us to evaluate the perceptual threshold of whisker-tactile inputs quantitatively. The perceptual threshold was decreased by the loss of vision at the juvenile period but not later, suggesting the presence of specific mechanisms underlying the cross-modal plasticity at this period.

## Supporting information

S1 Fig Sham tests. **A-C**, Sample raster plots of the licking events before and 5 s after the onset of LED stimulus cues (blue vertical line), the WT/reward (A), ChR2/no light (B), ChR2/no reward (C) rats, respectively. The dot was colored in red when it was rewarded, and in green for the first lick without reward. **D-F**, Histograms of licking probability before and 5 s after the onset of LED stimulus cues (blue vertical line) of the same rats shown previously (A-C). **G**, Cumulative probability plots of the reaction time. The vertical broken lines was drawn at 1 s while the horizontal ones at 0.75. **H**, Summary of the agility, the WT/reward (n = 6), ChR2/no light (n = 6), ChR2/no reward (n = 5) groups. **I**, Summary of the success rate, the WT/reward (n = 6), ChR2/no light (n = 6), ChR2/no reward (n = 5) groups. In A-F, each magenta broken line was drawn at 1 s after the cue stimulus.
(PDF)

S2 Fig Near-threshold task performance under test 2 (fixed LED duration at 0.6 ms). **A**, Sample raster plots of the licking events of a control rat aligned to the LED cues (blue vertical line) with a LED pulse duration (*t*_p_, ms) at 0.6. The power (*I*_p_, mW/mm^2^) of light was decreased from 2.9 (top), 1.28 (2nd row), 0.48 (3rd row) to 0.26 (bottom). **B**, Similar to A, but of an early VD rat. **C**, Similar to A, but of a late VD rat. **D-F**, Licking probability histograms of the above data shown in A-C, respectively. **G-I**, Cumulative probability plots of the reaction time of the same data shown in A-C, respectively. In A-F, each magenta broken line was drawn at 1 s after the cue stimulus.
(PDF)

S3 Fig Near-threshold task performance under test 3 (fixed power at 2.9 mW/mm^2^). **A**, Sample raster plots of the licking events of a control rat aligned to the LED cues (blue vertical line) with a LED power (*I*_p_, mW/mm^2^) at 2.9. The light pulse duration (*t*_p_, ms) was decreased from 1.0 (top), 0.5 (2nd row), 0.2 (3rd row) to 0.1 (bottom). **B**, Similar to A, but of an early VD rat. **C**, Similar to A, but of a late VD rat. **D-F**, Licking probability histograms of the above data shown in A-C, respectively. **G-I**, Cumulative probability plots of the reaction time of the same data shown in A-C, respectively. In A-F, each magenta broken line was drawn at 1 s after the cue stimulus.
(PDF)

S4 Fig Near-threshold task performance under test 4 (fixed power at 1.56 mW/mm^2^). **A**, Sample raster plots of the licking events of a control rat aligned to the LED cues (blue vertical line) with a LED power (*I*_p_ mW/mm^2^) at 1.56. The light pulse duration (*t*_p_, ms) was decreased from 1.0 (top), 0.5 (2nd row), 0.2 (3rd row) to 0.1 (bottom). **B**, Similar to A, but of an early VD rat. **C**, Similar to A, but of a late VD rat. **D-F**, Licking probability histograms of the above data shown in A-C, respectively. **G-I**, Cumulative probability plots of the reaction time of the same data shown in A-C, respectively. In A-F, each magenta broken line was drawn at 1 s after the cue stimulus.
(PDF)

S5 Fig Salient stimulus task, long test. The variations in the agility during salient LED stimulus task, 16 sets in each rat.
(PDF)

S6 Fig Estimation of the perceptual threshold using agility. Perceptual threshold was defined as the power (*I*_p_, mW/mm^2^) or the duration (*t*_p_, ms) of the LED cue pulse that changed the sign of agility from negative to positive. Practically, it was estimated from the agility (y)-*I*_p_ (x) or the agility (y)-*t*_p_ (x) relationship as the x-interception of a log-linear line connecting the point of negative agility with maximal *I*_p_ (or *t*_p_) and that of positive agility with minimal *I*_p_ (or *t*_p_).
(PDF)
