# Supplementary material for: Quantitative study of the somatosensory sensitization underlying cross-modal plasticity
Source: PLoS One. 2018 Dec 5;13(12):e0208089. doi: 10.1371/journal.pone.0208089 (PMC6281227; doi:10.1371/journal.pone.0208089)
Supplement: S1 Fig — A-C, Sample raster plots of the licking events before and 5 s after the onset of LED stimulus cues (blue vertical line), the WT/reward (A), ChR2/no light (B), ChR2/no reward (C) rats, respectively. The dot was colored in red when it was rewarded, and in green for the first lick without reward. D-F, Histograms of licking probability before and 5 s after the onset of LED stimulus cues (blue vertical line) of the same rats shown previously (A-C). G, Cumulative probability plots of the reaction time. The vertical broken lines was drawn at 1 s while the horizontal ones at 0.75. H, Summary of the agility, the WT/reward (n = 6), ChR2/no light (n = 6), ChR2/no reward (n = 5) groups. I, Summary of the success rate, the WT/reward (n = 6), ChR2/no light (n = 6), ChR2/no reward (n = 5) groups. In A-F, each magenta broken line was drawn at 1 s after the cue stimulus. (PDF) [file pone.0208089.s001.pdf]

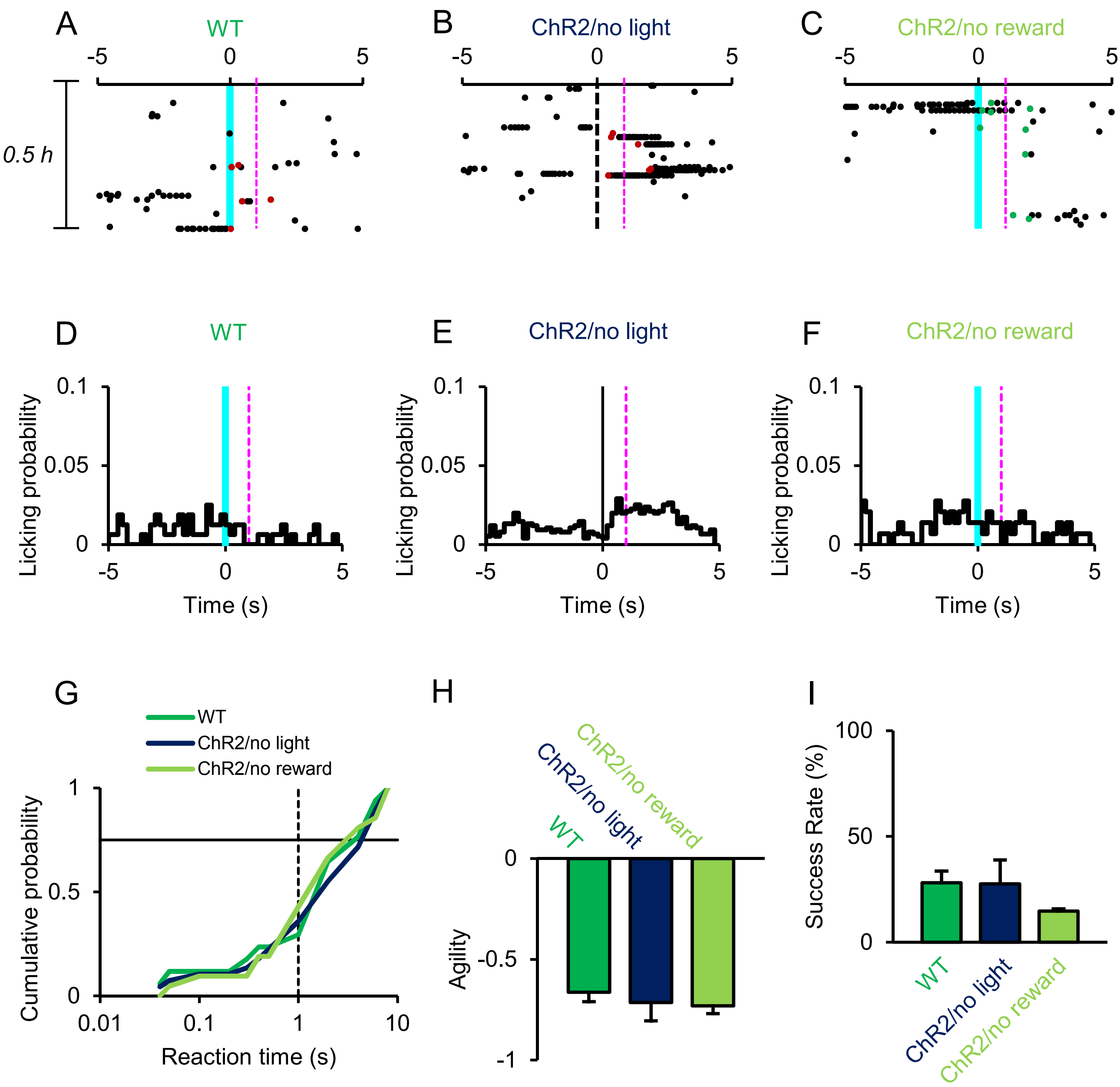

S1 Fig. Sham tests. A-C, Sample raster plots of the licking events before and 5 s after the onset of LED stimulus cues (blue vertical line), the WT/reward (A), ChR2/no light (B), ChR2/no reward (C) rats, respectively. The dot was colored in red when it was rewarded, and in green for the first lick without reward. **D-F**, Histograms of licking probability before and 5 s after the onset of LED stimulus cues (blue vertical line) of the same rats shown previously (A-C). **G**, Cumulative probability plots of the reaction time. The vertical broken lines was drawn at 1 s while the horizontal ones at 0.75. **H**, Summary of the agility, the WT/reward (n = 6), ChR2/no light (n = 6), ChR2/no reward (n = 5) groups. **I**, Summary of the success rate, the WT/reward (n = 6), ChR2/no light (n = 6), ChR2/no reward (n = 5) groups. In A-F, each magenta broken line was drawn at 1 s after the cue stimulus.
