# Supplementary material for: Quantitative study of the somatosensory sensitization underlying cross-modal plasticity
Source: PLoS One. 2018 Dec 5;13(12):e0208089. doi: 10.1371/journal.pone.0208089 (PMC6281227; doi:10.1371/journal.pone.0208089)
Supplement: S4 Fig — A, Sample raster plots of the licking events of a control rat aligned to the LED cues (blue vertical line) with a LED power (Ip mW/mm2) at 1.56. The light pulse duration (tp, ms) was decreased from 1.0 (top), 0.5 (2nd row), 0.2 (3rd row) to 0.1 (bottom). B, Similar to A, but of an early VD rat. C, Similar to A, but of a late VD rat. D-F, Licking probability histograms of the above data shown in A-C, respectively. G-I, Cumulative probability plots of the reaction time of the same data shown in A-C, respectively. In A-F, each magenta broken line was drawn at 1 s after the cue stimulus. (PDF) [file pone.0208089.s004.pdf]

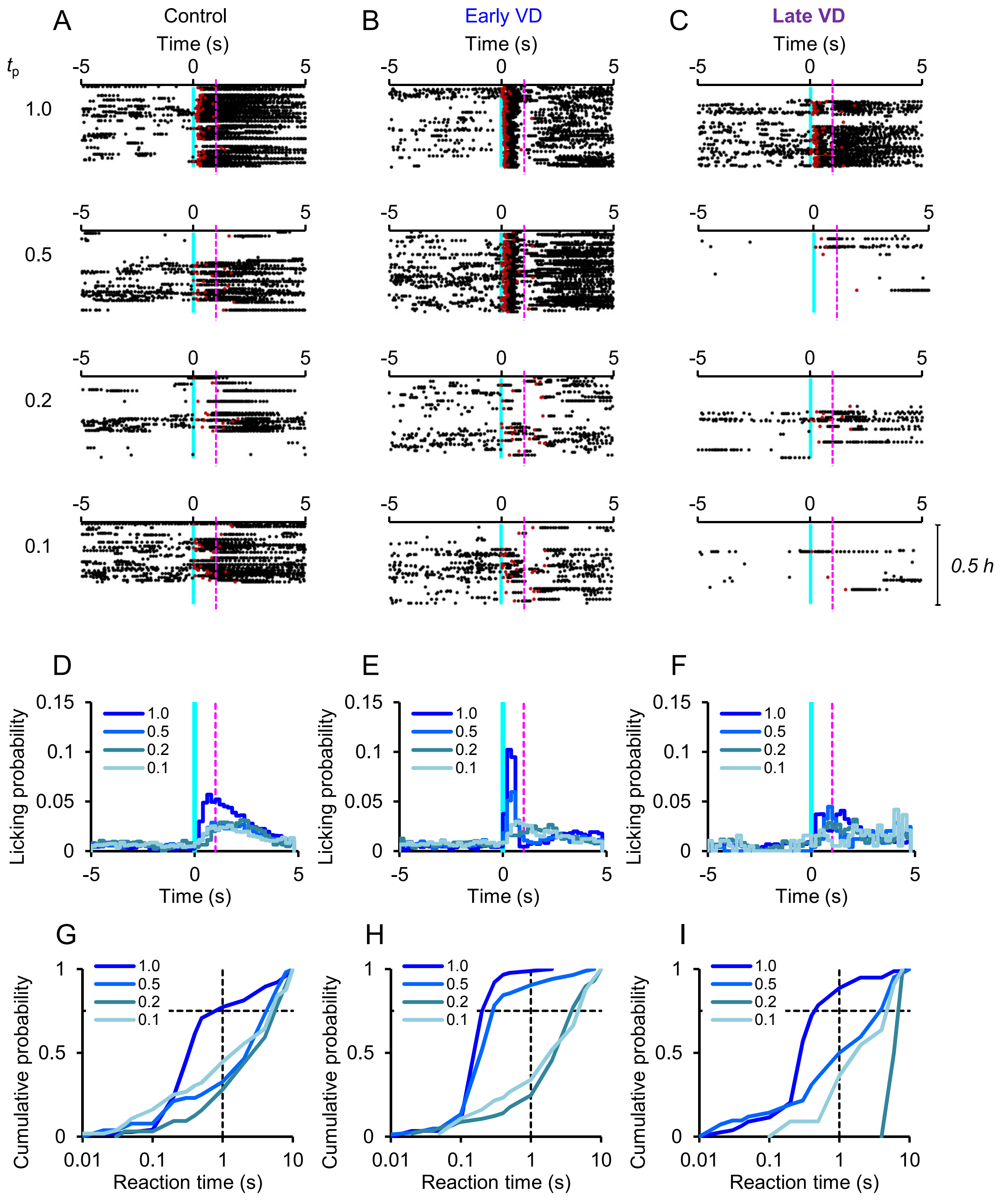

S4 Fig. Near-threshold task performance under test 4 (fixed power at 1.56 mW/mm²). A, Sample raster plots of the licking events of a control rat aligned to the LED cues (blue vertical line) with a LED power (I_p, mW/mm²) at 1.56. The light pulse duration (t_p, ms) was decreased from 1.0 (top), 0.5 (2nd row), 0.2 (3rd row) to 0.1 (bottom). **B,** Similar to A, but of an early VD rat. **C,** Similar to A, but of a late VD rat. **D-F**, Licking probability histograms of the above data shown in A-C, respectively. **G-I**, Cumulative probability plots of the reaction time of the same data shown in A-C, respectively. In A-F, each magenta broken line was drawn at 1 s after the cue stimulus.
