# Supplementary material for: Quantitative study of the somatosensory sensitization underlying cross-modal plasticity
Source: PLoS One. 2018 Dec 5;13(12):e0208089. doi: 10.1371/journal.pone.0208089 (PMC6281227; doi:10.1371/journal.pone.0208089)
Supplement: S5 Fig — The variations in the agility during salient LED stimulus task, 16 sets in each rat. (PDF) [file pone.0208089.s005.pdf]

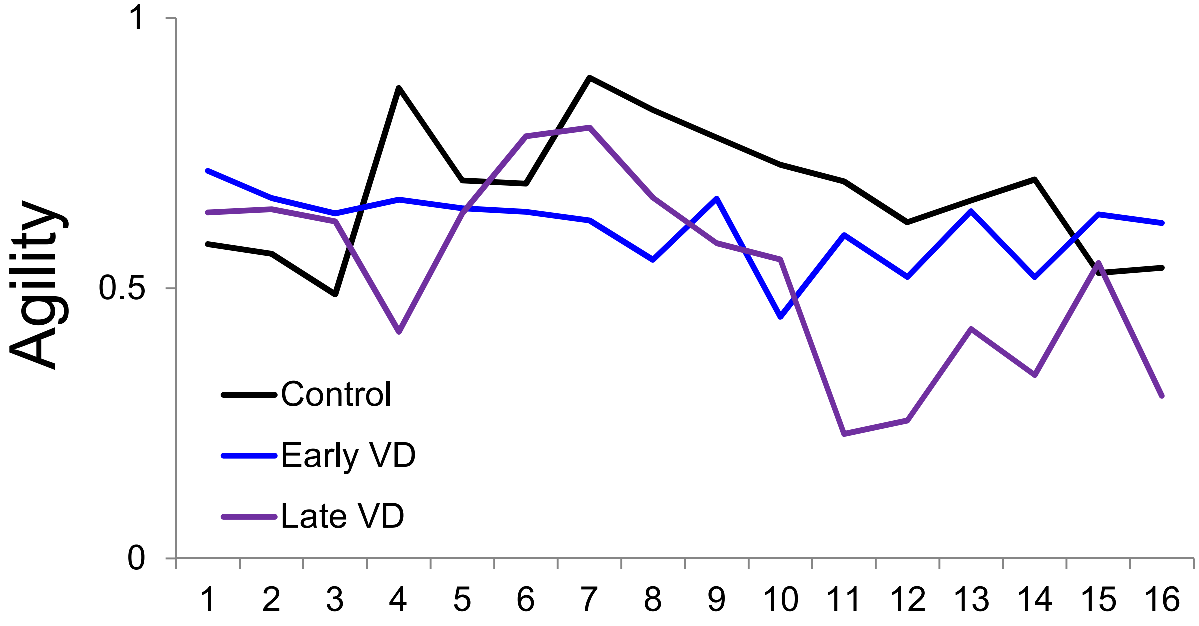

S5 Fig. Salient stimulus task, long test. The varies of agility when salient stimulus in each rat, 16 sets.
