# Supplementary material for: Quantitative study of the somatosensory sensitization underlying cross-modal plasticity
Source: PLoS One. 2018 Dec 5;13(12):e0208089. doi: 10.1371/journal.pone.0208089 (PMC6281227; doi:10.1371/journal.pone.0208089)
Supplement: S6 Fig — Perceptual threshold was defined as the power (Ip, mW/mm2) or the duration (tp, ms) of the LED cue pulse that changed the sign of agility from negative to positive. Practically, it was estimated from the agility (y)-Ip (x) or the agility (y)-tp (x) relationship as the x-interception of a log-linear line connecting the point of negative agility with maximal Ip (or tp) and that of positive agility with minimal Ip (or tp). (PDF) [file pone.0208089.s006.pdf]

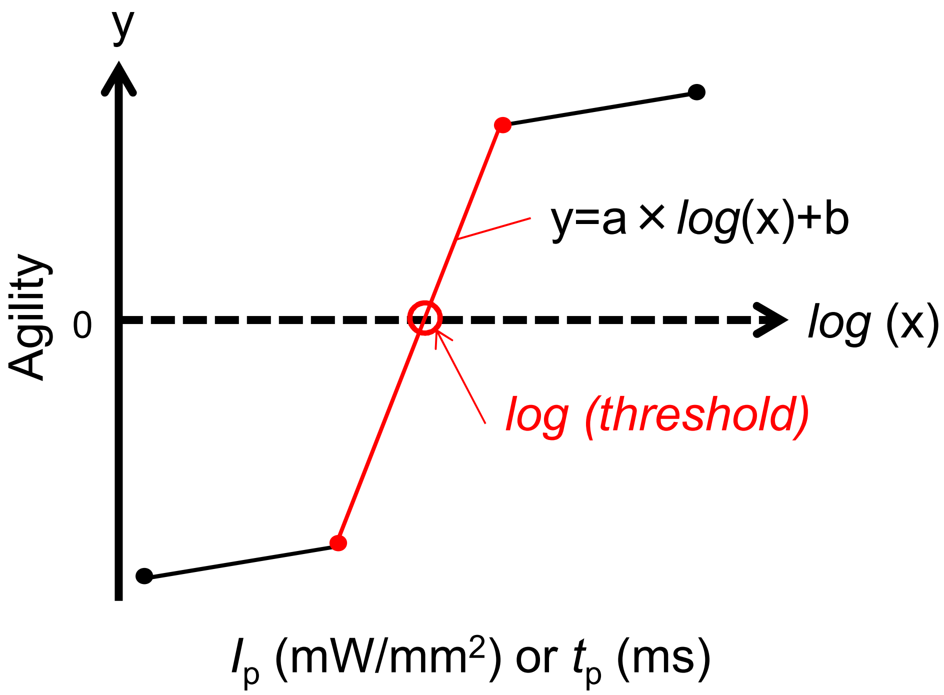

S6 Fig. Estimation of the perceptual threshold using agility. Perceptual threshold was defined as the power (I_p, mW/mm^2) or the duration (t_p, ms) of the LED cue pulse that change the sign of agility from negative to positive. Practically, it was estimated from the agility (y)-I_p (x) or the the agility (y)-t_p (x) relationship as the x-interception of a log-linear line connecting the point of negative agility with maximal I_p (or t_p) and that of positive agility with minimal I_p (or t_p).
